# Endoscopic gastric atrophy is strongly associated with gastric cancer development after *Helicobacter pylori* eradication

**DOI:** 10.1007/s00464-016-5211-4

**Published:** 2016-09-07

**Authors:** Osamu Toyoshima, Yutaka Yamaji, Shuntaro Yoshida, Shuhei Matsumoto, Hiroharu Yamashita, Takamitsu Kanazawa, Keisuke Hata

**Affiliations:** 1 Department of Gastroenterology, Toyoshima Endoscopy Clinic, 6-17-5 Seijo, Setagaya-ku, Tokyo, 157-0066 Japan; 2 Health Development Center, Tokyo Pharmaceutical Industry Health Insurance Society, Tokyo, Japan; 3 Department of Gastrointestinal Surgery, Graduate School of Medicine, The University of Tokyo, Tokyo, Japan; 4 Department of Surgical Oncology, Graduate School of Medicine, The University of Tokyo, Tokyo, Japan

**Keywords:** Stomach neoplasms, Risk factors, *Helicobacter pylori*, Chemoprevention, Atrophic gastritis, Endoscopy

## Abstract

**Background:**

Risk factors for gastric cancer during continuous infection with *Helicobacter pylori* have been well documented; however, little has been reported on the risk factors for primary gastric cancer after *H. pylori* eradication. We conducted a retrospective, endoscopy-based, long-term, large-cohort study to clarify the risk factors for gastric cancer following *H*. *pylori* eradication.

**Methods:**

Patients who achieved successful *H. pylori* eradication and periodically underwent esophagogastroduodenoscopy surveillance thereafter at Toyoshima Endoscopy Clinic were enrolled. The primary endpoint was the development of gastric cancer. Statistical analysis was performed using the Kaplan–Meier method and Cox’s proportional hazards models.

**Results:**

Gastric cancer developed in 15 of 1232 patients. The cumulative incidence rates were 1.0 % at 2 years, 2.6 % at 5 years, and 6.8 % at 10 years. Histology showed that all gastric cancers (17 lesions) in the 15 patients were of the intestinal type, within the mucosal layer, and <20 mm in diameter. Based on univariate analysis, older age and higher endoscopic grade of gastric atrophy were significantly associated with gastric cancer development after eradication of *H. pylori*, and gastric ulcers were marginally associated. Multivariate analysis identified higher grade of gastric atrophy (hazard ratio 1.77; 95 % confidence interval 1.12–2.78; *P* = 0.01) as the only independently associated parameter.

**Conclusions:**

Endoscopic gastric atrophy is a major risk factor for gastric cancer development after *H. pylori* eradication. Further long-term studies are required to determine whether *H. pylori* eradication leads to regression of *H. pylori*-related gastritis and reduces the risk of gastric cancer.

Gastric cancer is the third leading cause of death worldwide [1], and the numbers of cases and deaths are expected to rise as the world’s population increases and ages [2]. There is convincing evidence that chronic *Helicobacter pylori* (*H. pylori*) infection of the stomach provokes gastric cancer [3]. *H. pylori* has been estimated to be the cause of 89 % of non-cardiac gastric cancers [4]. A meta-analysis of randomized controlled trials reported a reduced risk of gastric cancer following *H. pylori* eradication therapy, with a relative risk of 0.66 (95 % CI 0.46–0.95) [5, 6].

The International Agency for Research on Cancer (IARC) Working Group Report in 2014 recommended that all countries explore the possibility of introducing population-based *H. pylori* screening and treatment programs as a strategy for gastric cancer prevention [2]. In Japan, national health insurance coverage was approved for eradication therapy in patients with endoscopically diagnosed chronic gastritis caused by *H. pylori* infection in February 2013 [2, 7].

Gastric cancer can develop even after eradication of *H. pylori*. If nationwide *H. pylori* treatment is conducted, then the majority of new gastric cancer cases will develop from “inactive” gastritis after *H. pylori* eradication. Identifying the characteristics and risk factors, other than active *H. pylori* infection, for gastric cancer that are not prevented by *H. pylori* eradication is thus key to designing strategies for controlling gastric cancer. The risk factors for gastric cancer during continuous infection with *H. pylori* have been well documented [2, 8–12], and several reports of metachronous gastric cancers after eradication in patients with endoscopically resected gastric cancer have been published [13–16]. However, little has been reported on the risk factors for primary gastric cancer after *H. pylori* eradication [17–19], especially in a large population of patients with plain chronic gastritis without peptic ulcers.

This study aimed to investigate the risk factors associated with primary gastric cancer after eradication of *H. pylori*. We conducted a retrospective, endoscopy-based, long-term cohort study involving a large cohort of patients with chronic gastritis without peptic ulcers and evaluated associations of various parameters with the risk of gastric cancer using multivariate analyses.

## Materials and Methods

### Patients

Patients who underwent esophagogastroduodenoscopy (EGD) at Toyoshima Endoscopy Clinic in Tokyo, Japan, between April 2002 and June 2014, were diagnosed with *H. pylori* infection, and achieved successful *H. pylori* eradication were included. These patients underwent EGD either for screening, a previous history of esophagogastroduodenal disease, present symptoms, abnormal findings by barium meal, or an abnormal serum pepsinogen level. Patients diagnosed as having gastric neoplasia (category 3, 4, or 5 according to the Vienna classification; i.e., noninvasive low-grade neoplasia, noninvasive high-grade neoplasia, or invasive neoplasia) [20] based on EGD at the time of enrollment were excluded. When lesions suspected to be gastric neoplasia were found but not determined histologically by EGD at the time of enrollment, the patients with those lesions were excluded if gastric neoplasia were confirmed within 1 year after *H. pylori* eradication. The other exclusion criteria were a past history of gastric neoplasia, previous gastrectomy, age younger than 20 years, or severe concomitant illness. Informed consent for each EGD and *H. pylori* eradication therapy was obtained from all patients.

The ethics review committees of external organizations approved the study protocol.

### Endoscopic findings and diagnosis of gastric cancer

EGD was performed by certificated endoscopists at Toyoshima Endoscopy Clinic using videoscopes (GIF-H240, GIF-H260, or GIF-HQ290, Olympus, Tokyo, Japan). Biopsy specimens were taken from lesions suspected to be gastric cancer or other major gastric findings and assessed histologically.

Histological evaluation was conducted according to the Vienna classification [20]. Gastric neoplasia was defined as category 3, 4, or 5 according to the Vienna classification (i.e., noninvasive low-grade neoplasia, noninvasive high-grade neoplasia, or invasive neoplasia). The diagnosis of gastric cancer was confirmed histologically using specimens from en bloc resection by endoscopy or surgery. Gastric cancer was defined as category 4 or 5 according to the Vienna classification (i.e., noninvasive high-grade neoplasia or invasive neoplasia). Lesions diagnosed as category 4 or 5 by biopsy were resected by endoscopy or surgery. Patients with category 3 lesions were recommended to undergo resection for precise diagnosis and to prevent progression to cancer. Based on the patient’s decision, the lesion was resected or followed up annually. Gastric cancer was classified according to Lauren as either the intestinal or diffuse type [21]. Patients with gastric or duodenal ulcer scars were also classified as having gastric or duodenal ulcers.

### Grade of gastric atrophy

**Fig. 1:**
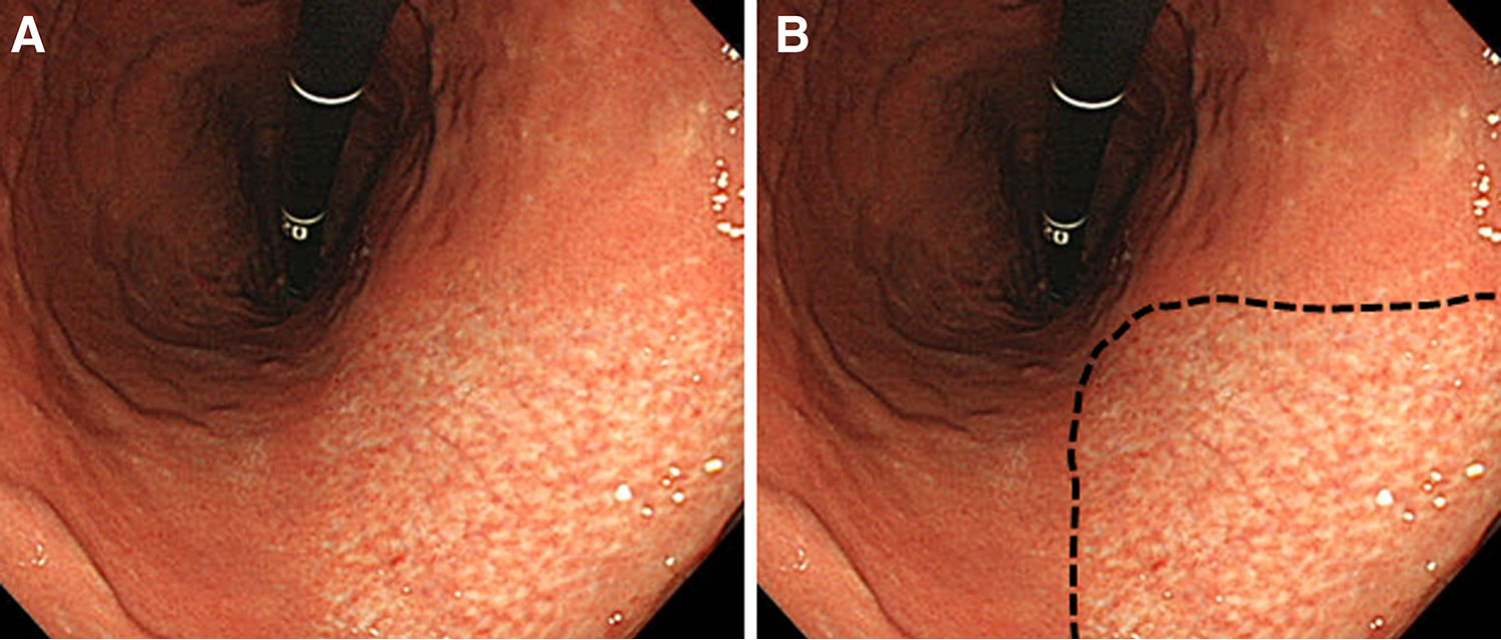
**A** A case of closed-type atrophy, Grade 3 (C-III): closed atrophy with the atrophic border recognized as an almost symmetrical enclosure and including the antrum and lesser curvature of the proximal gastric body. **B** The endoscopic-atrophic-border is indicated by a *dotted line*

**Fig. 2:**
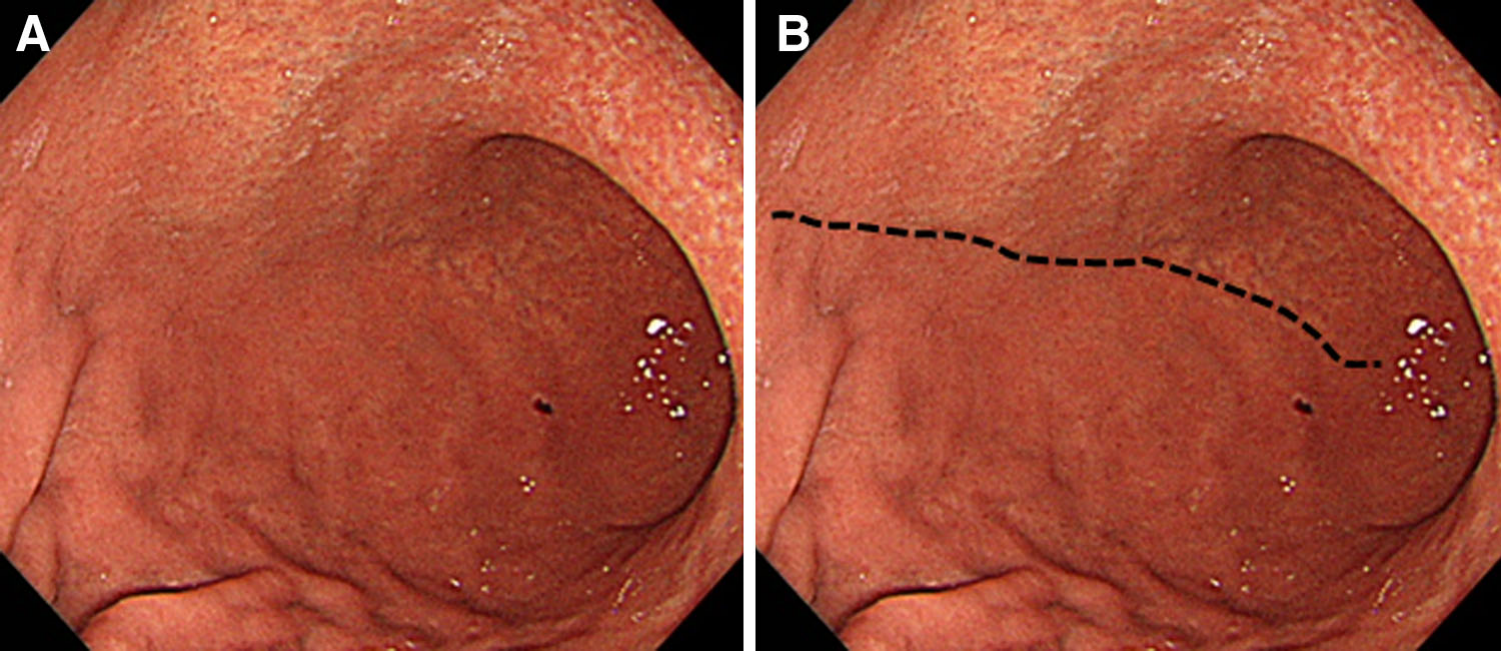
**A** A case of mild open-type atrophy, Grade 5 (O-II): open atrophy with the atrophic border lying amid the anterior wall. **B** The endoscopic-atrophic-border is indicated by a *dotted line*

**Fig. 3:**
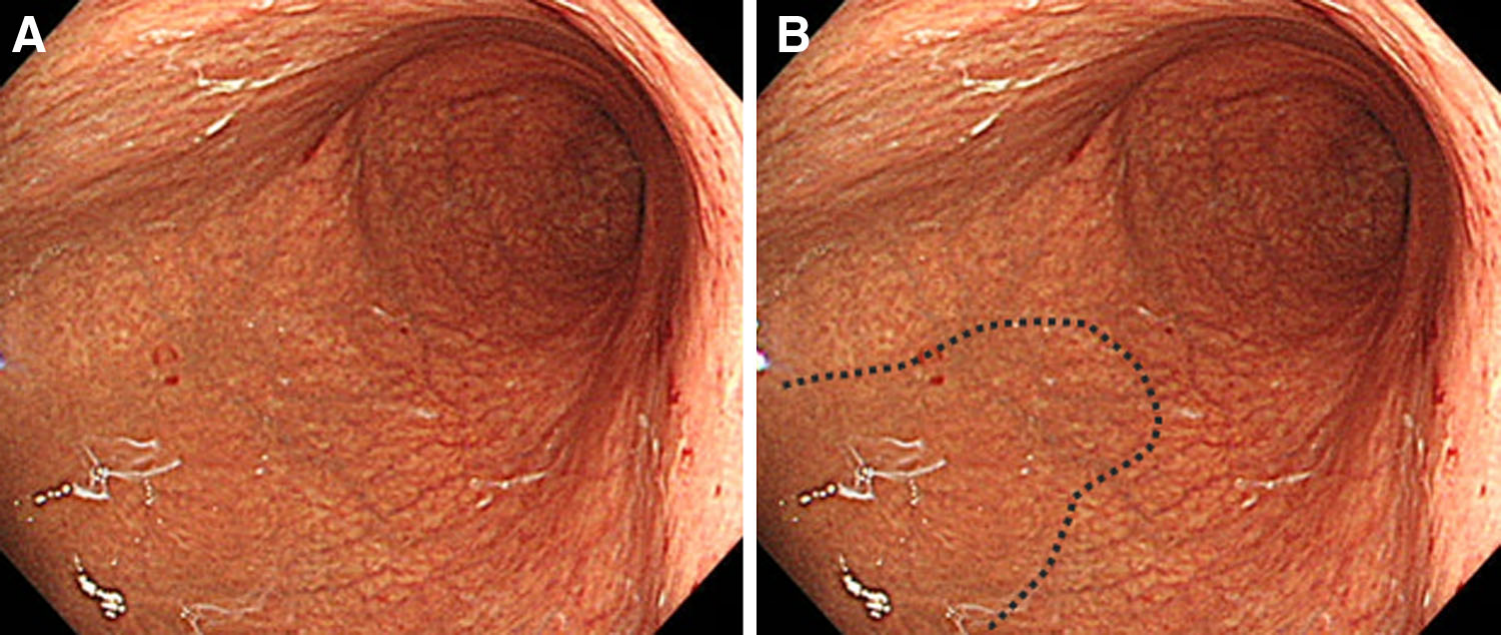
**A** A case of severe open-type atrophy, Grade 6 (O-III): open atrophy widely spread with the border between the anterior wall and the greater curvature. **B** The obscure endoscopic-atrophic-border is indicated by a *dotted line*

Gastric mucosal atrophy was evaluated according to the endoscopic-atrophic-border scale described by Kimura and Takemoto [22], which correlates with the results of histological evaluation [23]. They endoscopically categorized gastric atrophy as closed or open, where closed denoted mild and open denoted advanced atrophy. The endoscopic-atrophic-border was recognized as an almost symmetrical enclosure in the closed type, but not the open type. Each type of atrophy was also graded, where Grade 0 described no visible atrophy, Grade 1 (C-I) closed atrophy limited to the antrum, Grade 2 (C-II) closed atrophy limited to the antrum and lesser curvature of the distal gastric body, Grade 3 (C-III) closed atrophy including the antrum and lesser curvature of the proximal gastric body, Grade 4 (O-I) open atrophy with the atrophic border lying between the lesser curvature and the anterior wall, Grade 5 (O-II) open atrophy with the atrophic border lying amid the anterior wall, and Grade 6 (O-III) open atrophy widely spread with the border between the anterior wall and the greater curvature. Typical endoscopic images are shown in Figs. 1, 2, and 3.

### Diagnosis and treatment of *H. pylori* infection

*H. pylori* infection was diagnosed based on one or more positive results of the following *H. pylori* tests: ^13^C-urea breath test, serum anti-*H. pylori* antibody, *H. pylori* antigen in feces, or histology (hematoxylin and eosin, Giemsa, or immunologic staining).

All patients were informed of their infection status, and those with active *H. pylori* infection who consented to the eradication therapy received the following treatment regimens: lansoprazole (30 mg) or rabeprazole sodium (10 mg) together with amoxicillin (750 mg) and clarithromycin (200 or 400 mg) or metronidazole (250 mg) twice daily for 1 week. Participants in whom eradication treatment failed or who were allergic to penicillin were invited to undergo a second-line regimen: lansoprazole (30 mg) or rabeprazole sodium (10 mg) twice daily and a combination of amoxicillin (750 mg) and sitafloxacin (100 mg), or metronidazole (250 mg) and minomycin (100 mg) twice daily for 1 week, or amoxicillin (750 mg) twice and levofloxacin (500 mg) once daily for 10 days.

At least 8 weeks after the completion of eradication therapy, successful eradication was confirmed using one or two of the following measurements: negative ^13^C-urea breath test at two weeks after cessation of maintenance therapy with acid suppressors or negative *H. pylori* antigen in feces.

### Outcome measurement

We performed annual EGD surveillance after eradication of *H. pylori* infection for early detection of gastric cancer. The primary endpoint was the development of gastric cancer, and data were censored at the date of the final EGD. The effects of the following factors on gastric cancer development after *H. pylori* eradication were evaluated: sex, age, family history of gastric cancer, body mass index, drinking (every day or less), smoking (current or not), gastric ulcer, duodenal ulcer, and grade of gastric atrophy.

### Statistical analysis

Cumulative incidence rates of gastric cancer development were estimated using the Kaplan–Meier method. Kaplan–Meier curves were compared between gastric atrophy groups by the log-rank test. Hazard ratios (HRs) with 95 % CIs were calculated using Cox’s proportional hazards models in univariate and multivariate analyses. A multivariate analysis was performed for factors with *P* values <0.1 in univariate analyses. All statistical analyses were performed using IBM SPSS version 21.0 (IBM SPSS, Armonk, NY). All *P* values were two-sided; significance was indicated by a *P* value of less than 0.05.

## Results

Of the 1270 patients that fulfilled our inclusion criteria, 8 with gastric cancer (category 5: five patients, category 4: three) at the time of enrollment were excluded. In addition, four patients with lesions suspicious for gastric neoplasms were excluded because these lesions were confirmed to be gastric cancer (category 5: three, category 4: one) within 1 year after *H. pylori* eradication. Nineteen patients were excluded because they had a past history of gastric neoplasm (18 with category 4 or 5, one with category 3), 6 patients because they had undergone gastrectomy for peptic ulcer, and one patient because he was under 20 years old. Consequently, in total 1232 patients were analyzed.

The demographic characteristics of eligible patients are shown in Table 1. The mean age was 54.1 years; 661 patients were female; and 571 were male. Of them, 208 had gastric ulcer, 229 had duodenal ulcers, 53 had both gastric and duodenal ulcers, and 848 had only gastritis. Five hundred and seventy-eight patients had closed-type atrophy (Grade 0: 38, Grade 1: 68, Grade 2: 311, and Grade 3: 161) and 654 had open-type atrophy (Grade 4: 216, Grade 5: 162, and Grade 6: 276).

**Table 1:** Demographic characteristics of patients

Characteristics		All patients (*N* = 1232)	Gastric cancer development (*N* = 15)	No gastric cancer development (*N* = 1217)	*p* value^a^
Sex	Female/male	661/571	7/8	654/563	0.59
Age		54.1 ± 13	65.1 ± 8.5	54.0 ± 13.0	**0.0009**
Family history of gastric cancer	Yes/no	211/1021	5/10	206/1011	0.16
Body mass index		22.1 ± 3.1	23.1 ± 3.2	22.1 ± 3.1	0.2
Drinking	Yes/no	626/606	8/7	618/599	0.84
Smoking	Yes/no	353/879	1/14	352/865	0.08
Gastric ulcer	Yes/no	208/1024	5/10	203/1014	0.15
Duodenal ulcer	Yes/no	229/1003	1/14	228/989	0.33
Grade of gastric atrophy	0	38	0	38	**0.0003**
	1	68	0	68	
	2	311	0	311	
	3	161	1	160	
	4	216	3	213	
	5	162	2	160	
	6	276	9	267	

Bold values indicate statistical significance (*p* < 0.05)

^a^ *p* values derived from the Chi-squared tests, Student *t* tests, Fisher’s exact tests, and Mann–Whitney *U* tests as appropriate

**Fig. 4:**
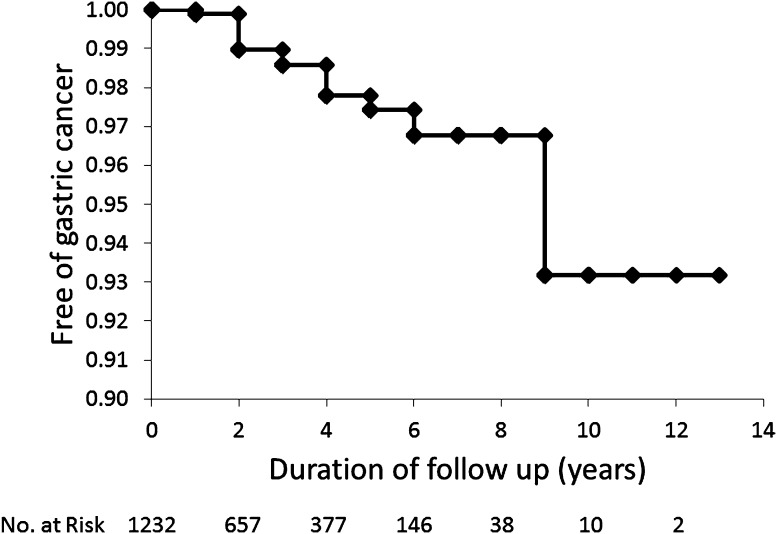
Kaplan–Meier analysis of the proportion of patients who remained free of gastric cancer

**Fig. 5:**
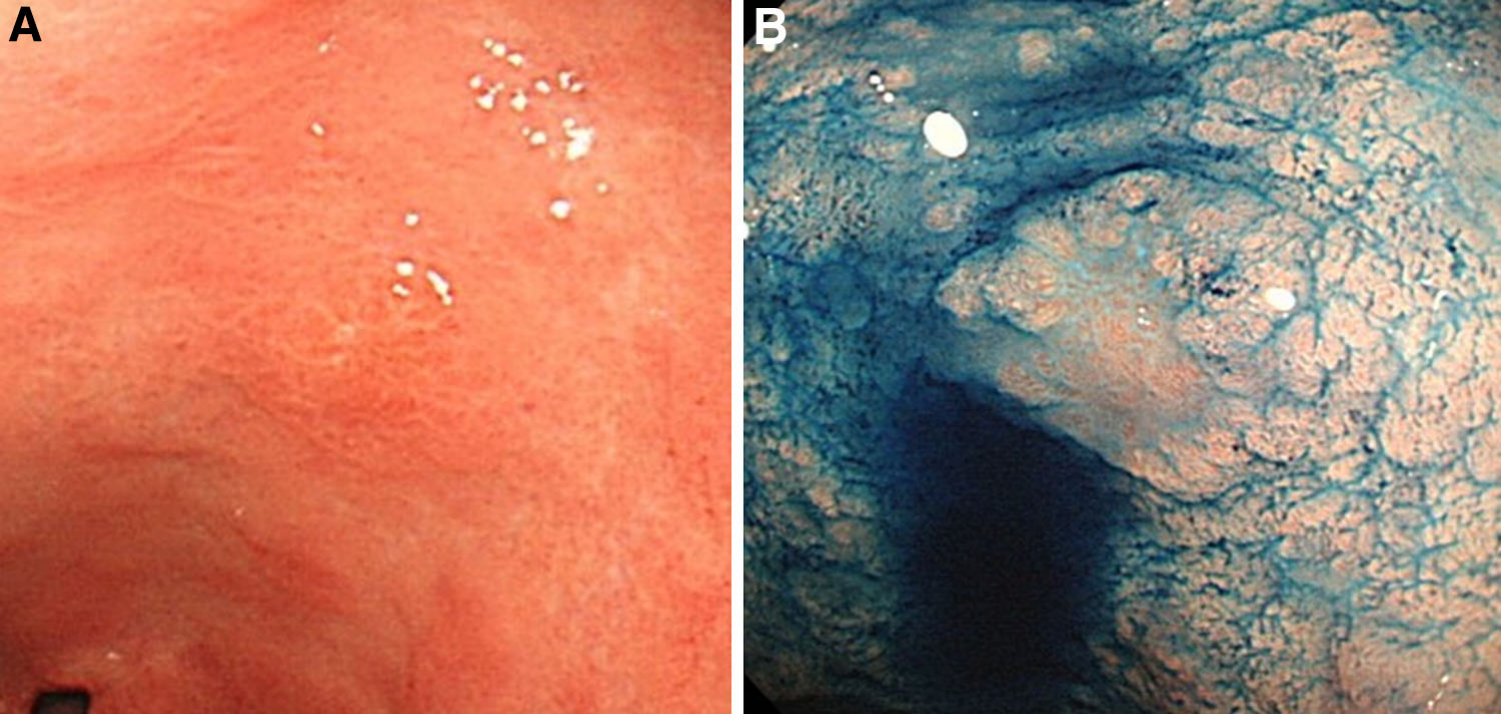
Endoscopic images of a typical case of gastric cancer. **A** White-light imaging. **B** White-light imaging with indigo carmine dye application

During the follow-up period, gastric cancer developed in 15 of 1232 patients after eradication of *H. pylori* infection (mean follow-up duration 2.46 years). The cumulative incidence rates of gastric cancer were 1.0 % at 2 years, 2.6 % at 5 years, and 6.8 % at 10 years (Fig. 4). All gastric cancers (17 lesions) that developed in the 15 patients were of the intestinal type based on Lauren’s classification, within the mucosal layer, and <20 mm in diameter. Endoscopic images of a typical case of gastric cancer are shown in Fig. 5.

Among the 17 lesions, nine were classified as category 5 and eight as category 4. One patient had two lesions of category 5, and another patient had one category 5 and one category 4 lesion simultaneously. There were some discrepancies in category between biopsy specimens and those obtained by en bloc resection. Five lesions classified as category 4 by biopsy analysis were diagnosed as category 5 following resection, and three determined to be category 3 by biopsy were corrected to category 4. Two patients with lesions diagnosed as category 3 by biopsy did not agree to undergo resection. These patients were followed up by annual EGD; one lesion retained a diagnosis of category 3 and the other became undetectable, though it may be resectable by repeated cold biopsies. These two cases were categorized into the no cancer development group, and data were censored at the time of the final EGDs.

By univariate analysis using Cox’s proportional hazards model, older age (*P* = 0.006) and higher grade of gastric atrophy (*P* = 0.002) were significantly associated, and gastric ulcer (*P* = 0.09) was marginally associated, with gastric cancer development after eradication of *H. pylori* infection (Table 2). A multivariate analysis was performed incorporating the parameters with *P* values <0.1 in the univariate analysis. This identified higher grade of gastric atrophy (HR 1.77; 95 % CI 1.12–2.78; *P* = 0.01) as the only parameter independently associated with development of gastric cancer after eradication (Table 3).

**Table 2:** Univariate analysis of factors associated with gastric cancer development after eradication of *H. pylori* infection (Cox’s proportional hazards model)

Factors		Hazard ratio	*p* value
Sex	Male	1.23	0.68
Age	Per 1 year	**1.07**	**0.006**
Family history of gastric cancer	Yes	1.90	0.24
Body mass index	Per 1 kg/m^2^	1.09	0.28
Drinking	Yes	1.02	0.96
Smoking	Yes	0.22	0.15
Gastric ulcer	Yes	2.53	0.09
Duodenal ulcer	Yes	0.34	0.29
Grade of gastric atrophy	Per 1 rank	**1.97**	**0.002**

Bold values indicate statistical significance (*p* < 0.05)

**Table 3:** Multivariate analysis of factors associated with gastric cancer development after eradication of *H. pylori* infection (Cox’s proportional hazards model)

Factors		Hazard ratio	95 % CI	*p* value
Age	Per 1 year	1.05	0.99–1.10	0.10
Gastric ulcer	Yes	2.43	0.82–7.26	0.11
Grade of gastric atrophy	Per 1 rank	**1.77**	1.12–2.78	**0.01**

Bold values indicate statistical significance (*p* < 0.05)

*CI* confidence interval

**Fig. 6:**
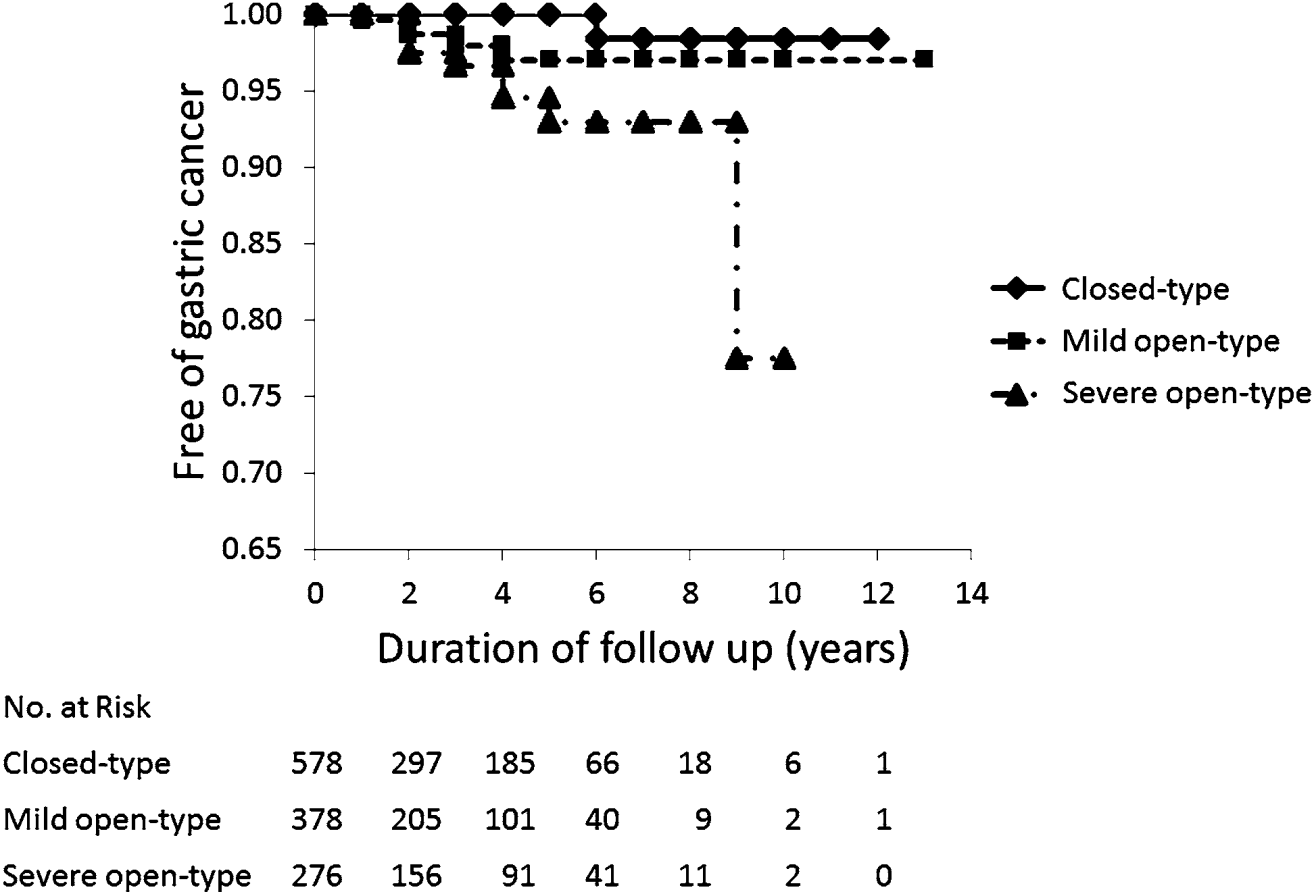
Kaplan–Meier analysis of the proportion of patients who remained free of gastric cancer stratified by the grade of gastric mucosal atrophy

The cumulative incidence rates of gastric cancer in closed-type atrophy (Grade 0–3), mild open-type atrophy (Grade 4 or 5; *P* = 0.02 vs. closed-type atrophy), and severe open-type atrophy (Grade 6; *P* = 0.0002 vs. closed-type atrophy) are shown in Fig. 6.

## Discussion

In this study, a higher grade of gastric atrophy was the only parameter independently associated with gastric cancer development after eradication of *H. pylori*. Although older age and gastric ulcer were associated in univariate analyses (older age significantly and ulcer marginally), they were not significant in the multivariate analysis.

Several studies have reported on patients with endoscopically resected gastric cancer, and two regarded gastric atrophy to be a risk factor for metachronous gastric cancer even after *H. pylori* eradication [13, 14]. On the other hand, reports on the risk factors for primary gastric cancer after *H. pylori* eradication, especially in a large population of plain chronic gastritis patients without peptic ulcers, are limited. Kamada et al. [17] reported in their cohort study of 1787 patients, including 453 with plain atrophic gastritis, that all patients in whom gastric cancer developed after *H. pylori* eradication had severe histological atrophy in the corpus. Take et al. [18] reported in their cohort study of patients with peptic ulcer diseases that persistent *H. pylori* infection, advanced gastric mucosal atrophy, and old age were significant risk factors for gastric cancer development after *H. pylori* eradication therapy. Kodama et al. [19] reported in their case–control study that patients who developed gastric cancer had a higher level of gastric atrophy and intestinal metaplasia than those who did not. In patients with continuous *H. pylori* infection, several risk factors have been identified as important parameters, including gastric atrophy. Age, gastric atrophy, intestinal metaplasia, gastric ulcer, absence of duodenal ulcer, drinking, and smoking habits have also been investigated [8–12]. Although multiple factors were assessed in the present study, only gastric atrophy was independently associated with gastric cancer development. One of the main reasons may lie in the fact that eradication of *H. pylori* incompletely and heterogeneously reverses the changes induced by chronic *H. pylori* infection. Histological inflammation improves or even normalizes in a few months, while atrophy gradually improves in a few years; intestinal metaplasia has also been reported to improve in 10 years [12, 24–26]. *H. pylori* eradication leads to normalization of gastric acid secretion in patients without severe atrophy [27], and improvement of expression of sonic hedgehog and CDX2 in mucosa with incomplete intestinal metaplasia [28], but MLH1 methylation is not affected in patients with intestinal metaplasia [29]. Wong et al. [9] and Yanaoka et al. [30] suggested that the effect of *H. pylori* eradication on reducing gastric cancer development would be greater for patients at an earlier stage of the gastric cancer cascade than those in later phases with advanced gastric atrophy or metaplasia. Therefore, gastric atrophy may be an important risk factor for gastric cancer development after eradication of *H. pylori* infection.

Intestinal-type gastric cancer develops mainly through the atrophy–metaplasia–dysplasia–cancer sequence [31]. On the other hand, inflammation induced by *H. pylori* can directly generate diffuse-type cancer from the non-atrophic stomach [32]. After *H. pylori* eradication, histological inflammation improves, rugal hyperplasia markedly regresses, and diffuse-type cancer development appears to be suppressed, according to a study by Watanabe et al. [33]. Indeed, in the present study, all 17 cancers in the 15 patients were of intestinal type. Diffuse-type cancer may be prevented within a short period after eradication of *H. pylori*, while the intestinal type continues to develop at a considerable rate for more than 10 years after eradication. The latter might start to regress when gastric atrophy and intestinal metaplasia begin to improve after a considerable period of time.

Generally, gastric cancer after eradication of *H. pylori* infection could include lesions missed at the time of eradication. In this study, we enrolled only patients in whom absence of neoplasm was confirmed by endoscopy. We also excluded patients with indeterminate lesions that were suspected to be gastric neoplasia and later confirmed as gastric cancer within 1 year after *H. pylori* eradication. The annual incidence rate of gastric cancer in this study averaged 0.5 % per year during the first 5 years, while the rate in the first year was 0.1 % per year, and the cumulative incidence rate was 1.0 % at 2 years. Several reports have suggested that *H. pylori* eradication might induce a flattened or dimmed appearance of some small gastric cancer lesions [34]. Therefore, some lesions may be difficult to detect after 1 year. Gastric cancers found in our cohort were all within the mucosal layer and <20 mm in diameter, suggesting that they were identified at an early stage. We attempted to conduct endoscopy as precisely as possible by routinely using sufficient sedation, contrasting the mucosa by dyeing with indigo carmine, and vigorously taking biopsies from small and depressed lesions, which have been reported to be characteristics of cancer after *H. pylori* eradication [17, 35]. The incidence rates of gastric cancer in the present study were higher than those in previous studies involving patients of similar age and *H. pylori* status [8–10, 17, 18, 30]. Our method of vigorous biopsies accompanied by dyeing might enable detection of smaller and more obscure lesions at an early stage.

There were several limitations to this study. First, the number of patients was too small for multiple factors to be evaluated fully. However, gastric mucosal atrophy identified by endoscopy was significantly associated with gastric cancer development after eradication of *H. pylori* infection. Second, we could not assess intestinal metaplasia. Histological intestinal metaplasia could also predict gastric cancer development [8, 36]. We did not routinely conduct biopsies of the background gastric mucosa in each patient. Given that progression of gastritis was the major risk factor for gastric cancer development even after eradication of *H. pylori*, the optimal indicator to evaluate gastritis should be determined. In particular, pathological indicators are likely to be informative and should be investigated in future studies. Third, Cag A status was not assessed. Because most *H. pylori* isolates from East Asia express the Cag A gene, our results indicate mainly the risk of infection with Cag A-positive species [2, 3]. Further analyses of other bacterial factors together with host genetic and environmental factors that modulate the response to *H. pylori* infection are warranted. Fourth, because ten endoscopists were engaged in this study, there might have been some variations in evaluations among the endoscopists. However, since all endoscopists were certified by the Japan Gastroenterological Endoscopy Society and familiar with endoscopic classification, endoscopies performed in the same patients were assumed to be generally congruent.

In conclusion, endoscopic gastric atrophy is a major risk factor for gastric cancer development after *H*. *pylori* eradication. Patients with advanced atrophy should be carefully monitored for at least 10 years. Further long-term studies are required to determine whether *H*. *pylori* eradication leads to regression of *H*. *pylori*-related gastritis and a reduced risk of gastric cancer.
